# Glucagon Secreting Cells Responds to Insulin Secretion *In vitro* Using Immunocytochemistry

**DOI:** 10.4103/0975-1483.63154

**Published:** 2010

**Authors:** MK Aswar, UM Aswar, NK Subhedar

**Affiliations:** 1 *Sinhgad Institute of Pharmacy, Narhe, Pune-41, India*; 1 *Bharati Vidyapeeth’s Poona College of Pharmacy, Erandwane, Pune, India*; 2 *Indian Institute of Science Education and Research, Pashan, Pune, India*

**Keywords:** Glucagon, immunocytochemistry, insulin

## Abstract

In the present study, pancreas of rats were dissected and transferred to HEPES buffer (25 mM, pH 7.4). The control tissue pieces were kept in culture medium for one hour and the treated tissues were kept in same medium for 30 minutes and incubated with Insulin (10 nm and 100 nm) for another half hour, then tissues were transferred to Bouin‘s fixative (overnight at 40 ° Cc), cryosectioned (15 µm at -16 0 c) and subjected to immunocytochemical labeling with antibodies against Glucagon.

**Results:**

In the sections of control tissue, the Glucagon Immunoreactive Cells (GIC) were distinctly visible; on average 40-50 cells were counted in each islet. However *in vitro* treatment with 10 nm insulin caused 285.89 % increase in the GIC and was found to be highly significant (*P*< 0.001). Whereas in 100 nm Insulin treatment, 206.41% increase in GIC was seen, this was significant with the control but non-significant with 10 nm Insulin treatment

## INTRODUCTION

The pancreas is an elongated, some-what flattened retroperitoneal gland attached to the duodenum by a duct which serves to transport its digestive secretions into the intestine. It contains two major types of secretory tissues, reflecting its dual function as an exocrine and endocrine gland. The exocrine part constitutes about 99% of total volume of cells that are arranged as small cluster called acini. The remaining 1% of the tissue constitutes the endocrine portion of the pancreas and said as Islets of Langerhans, each only about 0.3 mm in diameter[1]

The Islets of Langerhans contains three distinct types of hormone secreting cells viz. α-cells secreting Glucagon, β-cells secreting Insulin, and δ-cells secreting Somatostatin. The close interaction among these cell types in the Islets of Langerhans allows direct control on secretion of some of the hormones by other hormone involving paracrine control.[2] The glucagon secreted by α-cells of pancreatic islets is a member of the family of structurally related peptides that includes vasoactive intestinal peptide, gastric inhibitory peptide,[3] Secretin[4] and growth hormone releasing hormone.[5] In the pancreas, the glucagon is derived from preproglucagon, a 180 amino acids peptide precursor with a molecular weight of 3485 D and is composed of a single chain of 29 amino acids and is identical in all mammals.[6] It consists of five separately processed domains, an amino terminal signal peptide followed by glicentin-related pancreatic peptide, glucagon, glucagon-like peptide-I and glucagon-like peptide-II.[7]

The principal physiological action of glucagon is to increase blood glucose level when it falls below normal, mostly by its potent stimulation of hepatic glycogenolysis. This effect is mediated via stimulation of adenylyl cyclase and activation of cAMP- dependent protein kinase. Phosphorylase, the rate-limiting enzyme in glycogenolysis, is activated by glucagon as a result of cAMP- stimulated phosphorylation, while concurrent phosphorylation of glycogen synthase inactivates the enzyme; glycogenolysis is enhanced and glycogen synthesis is inhibited. It is interesting to note that glucagon has no effect on skeletal muscle glycogen, presumably because of the lack of glucagon receptors on skeletal muscle.[8] Apart from its hyperglycemic action, glucagon has a potent inotropic and chronotropic effect on the heart producing effect similar to -adrenoceptor agonist. In adipose tissue, glucagon stimulates adenylyl cyclase and increases lipolysis.[9]

The secretion of glucagon is regulated mainly by dietary glucose, insulin, amino acids, and fatty acids.[10] Glucose is one of the potent inhibitor of glucagon secretion. In vitro studies using isolated pancreatic islets from fed and from 4 and 8-day fasted rats revealed a continue increase of glucagon during prolonged fasting.[11] Studies also revealed that various forms of stress such as trauma or infection are associated with hyperglucagonemia.[12] As a part of “fight or flight” response, enhanced sympathetic outflow and increased secretion of catecholamine could stimulate glucagon secretion. The concomitant insulinopenia would also promote glucagon secretion and allow mobilization of amino acids and free fatty acids respectively from muscles and adipose tissues.[13] Immunoreactive Calcitonin gene- related peptide (CGRP) has also been shown to occur in the fibers innervating blood vessels and islets and plays a key role in the regulation of glucagon secretion in rats.[14,15] It has been reported that glucagon secretion increases during hypoxia.[16] In addition to above pharmacological agents, Noradrenaline, adrenaline, acetylcholine and GABA were also found to influence the secretion of glucagon from rat pancreatic α-cells.[17] Glucagon has emerged as an agent of considerable therapeutic value for the treatment of some diseases. The major use of glucagon is for emergency treatment of severe hypoglycemic reaction in insulin- dependent patients. Glucagon is sometime useful for reversing the cardiac effects of an overdose of beta- blockers as it increases cAMP production in the heart. Glucagon has also been used to relax the intestinal tract to facilitate radiographic ileography. Glucagon has been used for diagnostic purposes to distinguish obstructive form of hepatocellular jaundice.[18]

From the foregoing, it is clear that a number of factors are involved in the regulation of glucagon containing cells. However, these are based on a variety of techniques, and the available information does not permit unequivocal conclusions. In the present study, an attempt has been made to define the role of insulin in the regulation of glucagon secretion using an *in vitro* incubation system. The response of the glucagon cells was evaluated by selectively labeling the cells with antibodies against glucagon and processed for immunocytochemistry.

## MATERIALS AND METHODS

### Animals

Adult male Wistar rats weighing 175-200 g were used in the present study. They were housed in separate cages and maintained under natural photoperiodic conditions. Food and water were provided *ad libitum*. All the experiments were carried out between 9.00 to 11.00 hrs to avoid nyctohemeral influences, if any. The experiment protocol was approved by the Institutional Animal Ethical Committee of UDPS, Nagpur. The rats were anesthetized with chloroform and perfused transcardially with 200 ml of ice-cold phosphate buffered saline. The pancreatic tissue were dissected out and kept in HEPES buffered Hank‘s balanced salt solution supplemented with 10% BSA (bovine serum albumin, pH 7.4).

### *In vitro* static incubation system

The tissue slices were given three washes in HEPES (25 mM) medium and immediately transferred into small glass vials containing 1 ml of HEPES buffered Hanks balanced salt solution (HBSS; pH 7.4) supplemented with BSA and glucose (0.1% each). The tissue was kept in the static chamber that provides a constant temperature (17°C) and supply of 95% O_2_and 5% CO_2_. The control tissue fragments were incubated in the above medium for 60 minutes. The experimental tissue fragments were pre-incubated separately in normal medium for 30 minutes, followed by 30 minutes incubation in the same medium containing Insulin at 10 and 100 nM concentrations. At the end of 60 min incubation, the tissue slices were fixed in Bouin’s fixative and kept overnight in the same fixative at 40C. The tissues were then cryoprotected with 30% sucrose in phosphate buffer at 4°C overnight. The tissues were then rapidly frozen with expanding CO_2_and cut transverse plane at 15 µm thickness on a cryostat at -16°C. The sections were mounted on poly-L-lysine- coated slides, dried and processed for immunocytochemistry using Streptavidin-biotin method.

### Morphometric analysis

The islets of Langerhans of the control and treated samples were examined at 400X. Total of ten islets randomly chosen from each tissue sample were considered and the number of glucagon immunoreactive cells per islet was counted. The mean ± SE for each sample were calculated and the data was processed for statistical analysis and the difference between the control and experimental values was analyzed using Student’s t-Test and one-way Analysis of variance (ANOVA) followed by Tukey-Kramer multiple comparison test.

## RESULTS

The islets of Langerhans constitute the endocrine portion of the pancreas. In the freshly collected tissue from the animals as well as the fragments of pancreatic tissue maintained in culture for 60 minutes; glucagon immunoreactive cells appeared in cluster along the periphery of islets. Individual cells are prominent and show distinct nuclei. The cytoplasm in the cells is generally laden with immunoreactive material, no significant changes in the quality of immunoreactive or cytomorphological characteristics of cells could be noticed after incubation in the normal medium for 60 minutes. This indicates that the static incubation system employed herein can protect the cellular integrity and appears to be suitable for the short-term culture studies.

**Figure 1:**
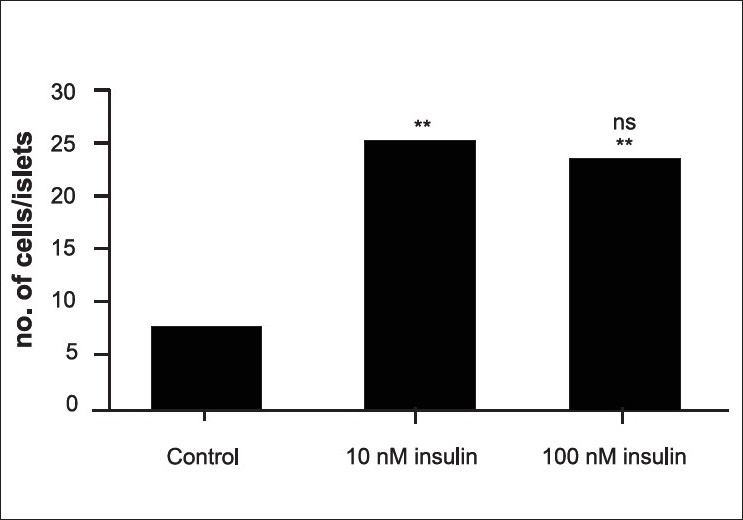
Effect of insulin treatment on the population of glucagon secreting cells. Statistical analysis was carried out using one way ANOVA followed by Tukey-Kramer multiple comparison test. **indicates p<0.001 compared to control, ns indicates p>0.05 compared to 10 nM insulin treatment.

**Figure 2:**
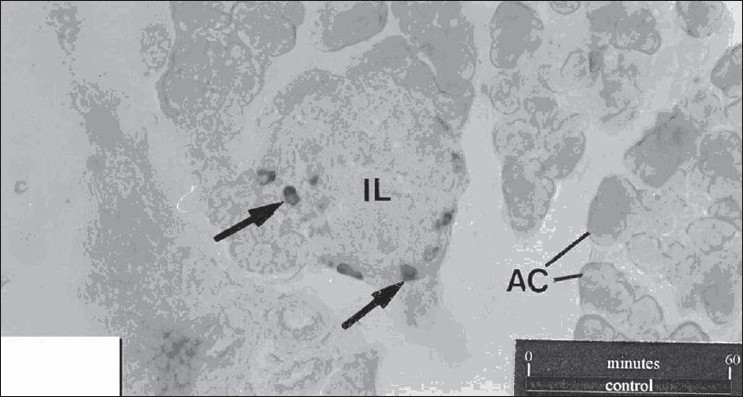
T.S. through the control pancreas of rat showing glucagons secreting cells (arrow) in the islets of Langerhans (IL) immunolabeled with polyclonal antibodies against human glucagons. AC, acinar cells. X280.

**Figure 3:**
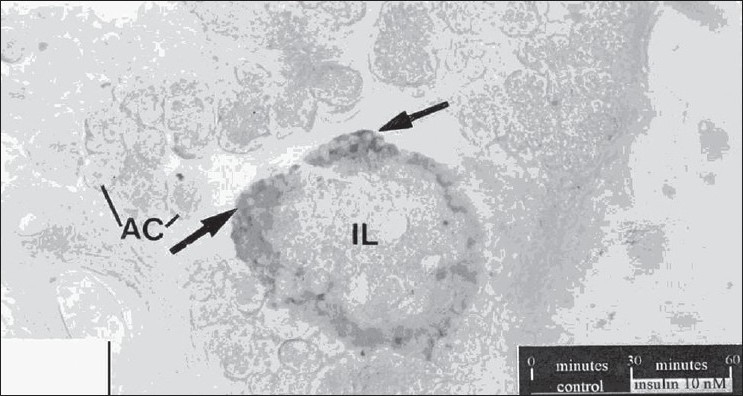
T.S. through the insulin treated (10 nM) pancreas sections. An increase in the number of immunoreactive cells can be seen. AC, acinar cells. X 280.

**Figure 4:**
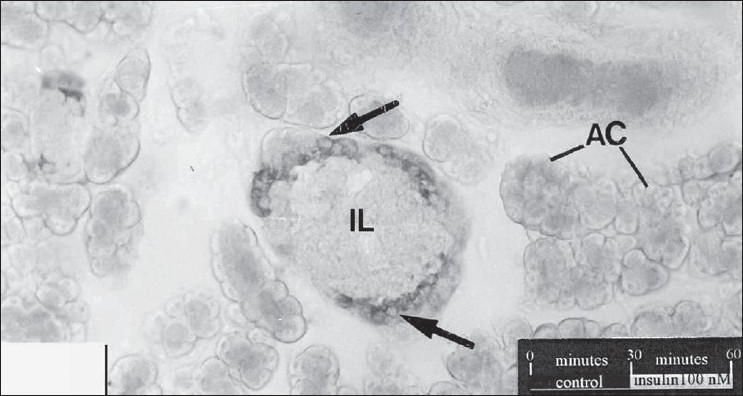
T.S. through the insulin-treated (100 nM) pancreas sections. An increase in the number of immunoreactive cells can be seen as compared to control. AC, acinar cells. X 280.

The results of the effect of insulin on the population of glucagon are summarized in Figure 1. In the sections of control tissue, a limited number of glucagon immunoreactive cells were seen along the periphery of islets of Langerhans [Figure 2]. While the treatment with 10 nM insulin caused a 285.89 % increase in the number of glucagon immunoreactive cells [Figure 3], which was found to be highly significant (P< 0.001), 206.41% increase was observed following insulin treatment at 100 nM [Figure 4]. This change was also found to be highly significant as compared to control. However, no significant difference was found between cell population in the tissue samples treated with 10 nM and 100 nM concentration of insulin.

## DISCUSSION

The *in vitro* technique was found invaluable in the present study on regulation of glucagon cells in the rat pancreas. The assembly for *in vitro* incubation was quite convenient to set up. Since the tissue were incubated in microtiter plates, a small quantity of the medium and micro quantities of dosages was required to conduct the study; this made the system economical and permitted testing of large number of sample in a reproducible manner. Further, *in vitro* system provided environment free from secondary effects e.g. hormonal, neural, etc. that are encountered in *in vivo* and confound the interpretation of the results. Influences for internal and external cyclical factors like the photoperiod, estrous, and temperature were also avoided. The *in vitro* approach enabled us to circumvent recurrent painful conditions generally encountered in *in vivo* chronic studies. In vitro incubation permits the drug to act directly on the tissue and greatly reduce the response time. In the present investigation, *in vitro* incubation could be sustained for a period of one hour without any detrimental effect on the tissue. No differences were seen in the immunocytochemical profile of the freshly sectioned tissue and the tissue incubated *in vitro* for one hour; the cellular as well as cell nuclear profiles were quite similar in both the preparations. This confirms the suitability of *in vitro* technique for the present study. Several workers have fruitfully employed the immunocytochemistry method to investigate the cellular response in pancreas and other tissue.[19–21] Some information on the role of insulin in regulating glucagon secreting cells is also available using *in vitro* studies; Maruyama *et al*. 1984 reported intrapancreatic insulin in rat exerted an ongoing release inhibiting action on the alpha cells. Insulin was found to inhibit glucagon secretion by paracrine Beta- cells activity in human.[22] Insulin was also found to inhibit the secretion of glucagon from chicken pancreas.[23] In the present study, the incubation of pancreatic tissue with insulin at both dose levels (10 and 100 nM) resulted in significant increase in the number of Glucagon containing cells, the response at lower dose was more prominent than at higher dose. It seems that insulin may be influencing the secretory but not synthetic component of the glucagon cells physiology. As a result the intracellular hormonal content might be getting augmented in the alpha- cells resulting in the overall increase in their population.
